# MircoRNAs as regulators of regulated cell death in cancer progression: a focus on ferroptosis, anoikis and pyroptosis

**DOI:** 10.3389/fphar.2026.1862213

**Published:** 2026-08-25

**Authors:** Di Xiao, Yanzhou Luo, Liyuan Liu, Yajie Lu, Xiaofang Xie, Zhaokai Zhou, Bin Ma, Fu Peng

**Affiliations:** 1 West China School of Pharmacy, Sichuan University, Chengdu, China; 2 State Key Laboratory of Southwestern Chinese Medicine Resources, Chinese Medicine Germplasm Resources Innovation and Effective Uses Key Laboratory of Sichuan Province, Chengdu University of Traditional Chinese Medicine, Chengdu, China; 3 Department of Urology, National Clinical Research Center for Metabolic Diseases, The Second Xiangya Hospital of Central South University, Changsha, Hunan, China; 4 Department of Pharmacology, Key Laboratory of Drug-Targeting and Drug Delivery System of the Education Ministry, Sichuan Engineering Laboratory for Plant-Sourced Drug and Sichuan Research Center for Drug Precision Industrial Technology, Chengdu, China

**Keywords:** anoikis, cancer, ferroptosis, MicroRNA, pyroptosis, regulated cell death

## Abstract

Cancer progression is shaped not only by genetic alterations but also by dynamic remodeling of regulated cell death. MicroRNAs (miRNAs) have emerged as central regulators of these processes by coordinating gene expression, metabolic balance, and stress responses. This review summarizes current understanding of how miRNAs modulate ferroptosis, anoikis, and pyroptosis across liver, lung, gastric, breast, and other cancers. They influence ferroptosis through lipid peroxidation and antioxidant systems, regulate anoikis via integrin signaling and cytoskeletal remodeling, and tune pyroptosis through inflammasome activation and gasdermin-mediated pore formation. Emerging evidence suggests that miRNAs function as integrative nodes linking metabolic reprogramming, immune-inflammatory signaling, and adaptive survival pathways. Their stability, druggability, and cross-pathway influence highlight their promise as biomarkers and therapeutic targets, and advancing RNA-based technologies may enable more precise manipulation of cell-death programs to overcome resistance and refine cancer treatment.

## 1 Introduction

Cancer represents one of the leading threats to human health worldwide (Fu et al., 2022). Its initiation and progression involve not only genetic mutations and aberrant signaling pathways, but also profound alterations in cell death programs. Traditionally, apoptosis is regarded as the predominant form of cell death, maintaining tissue homeostasis and preventing uncontrolled proliferation (D’Arcy, 2019). However, accumulating evidence demonstrates that cancer cells acquired survival advantages by evading or reprogramming death pathways, driving tumor initiation, progression, and therapeutic resistance (Fulda, 2009; Hanahan and Weinberg, 2011; Pistritto et al., 2016).

So far, cell death has been regarded as a vital process in cancer, shaping tumor progression while simultaneously serving as a central target for therapeutic intervention. Numerous novel forms of cell death in cancer are explored. In this context, the concept of regulated cell death (RCD) is proposed to encompass a spectrum of molecularly defined, druggable death modalities. Extensive efforts have focused on targeting these RCD pathways with synthetic small-molecule compounds (Liu W. et al., 2022) as well as plant-derived natural agents (Yang et al., 2023), underscoring their therapeutic potential across multiple death programs (An et al., 2026). However, therapeutic targeting of apoptosis has been impeded by frequent drug resistance in tumors, often due to mutations in apoptotic pathways or defective caspase activation (Singh et al., 2019; Pfeffer and Singh, 2018). This limitation spurs intense interest in other RCD pathways, which offer alternative, potentially more effective, strategies to eradicate apoptosis-resistant cancer cells and overcome treatment failures (Tang et al., 2019; Chen et al., 2022). Compared with classical apoptosis, these RCDs encompass a broader spectrum of death modalities that operates through diverse and intersecting molecular pathways. Different RCD types engage distinct executioners while sharing some upstream regulators, which play complex roles in cell elimination, immune response, metastasis, tumor microenvironment, etc. They act as tumor-suppressive mechanisms restricting cell survival. Ferroptosis further illustrates this, given its links to inflammatory microenvironments (Dou et al., 2022) and its ability to modulate immune activity within the tumor microenvironment (Zhu et al., 2023). However, cancer cells can also exploit their regulation to enhance resistance and invasiveness.

**FIGURE 1:**
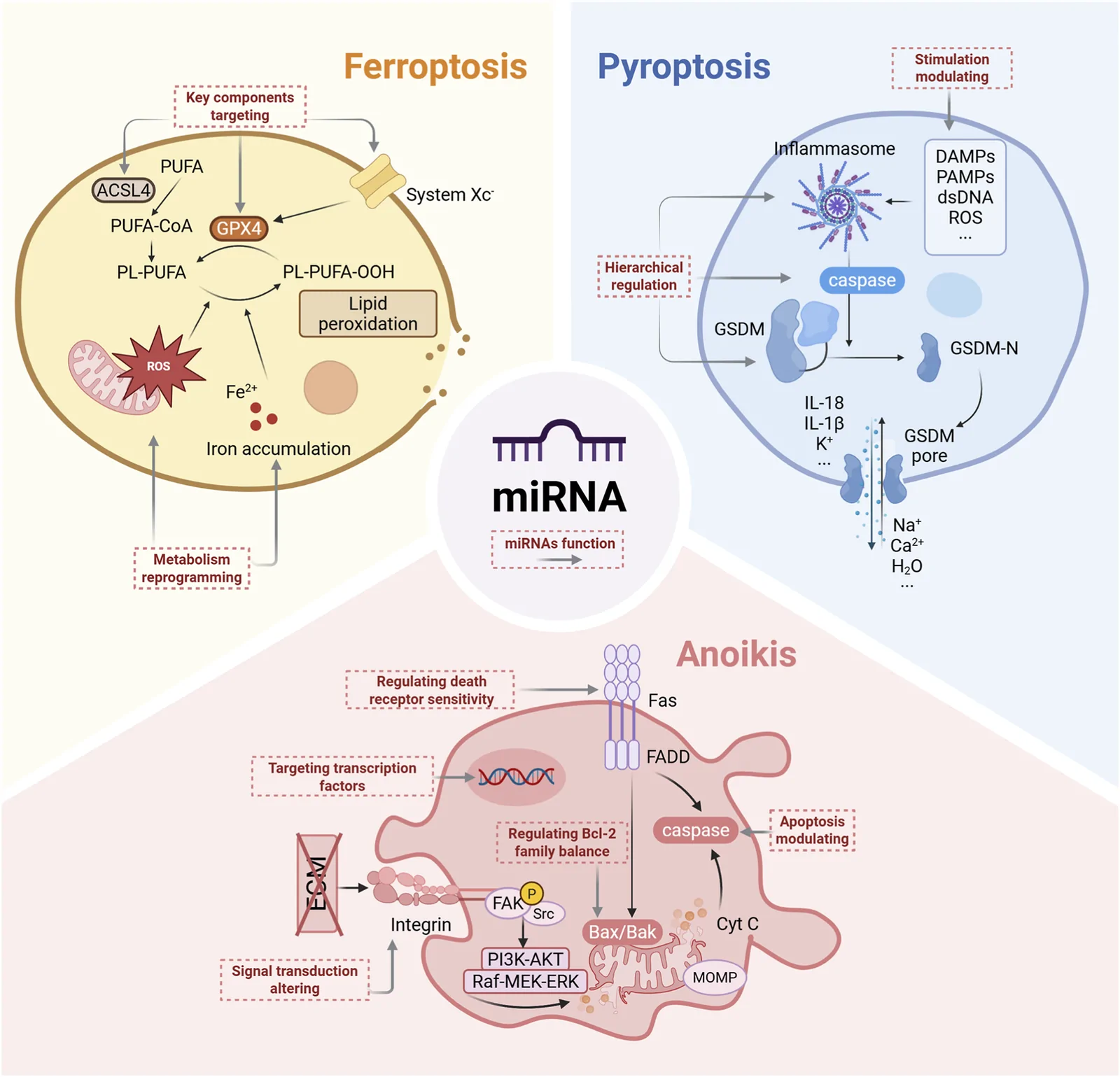
MiRNA-mediated regulation of three major forms of RCD in cancer. In ferroptosis, miRNAs regulate SLC7A11/GPX4 axis, iron handling, ROS accumulation and ACSL4-mediated lipid peroxidation, thereby shaping ferroptotic sensitivity and tumor progression. In anoikis, miRNAs enable cancer cells to evade apoptosis by remodeling adhesion signaling (e.g., Integrin/FAK-Src, PI3K-AKT, Raf-MEK-ERK), modulating Bcl-2 family balance and death receptors (e.g., Fas), and targeting key transcription factors to block apoptotic execution. In pyroptosis, miRNAs modulate upstream stimulatory signals including ROS and DAMPs, as well as pivotal molecules within the subsequent inflammasome-caspase-GSDM pyroptotic pathway.

Among the diverse forms of RCDs, ferroptosis, anoikis, and pyroptosis have become subjects of intense research in recent years (Figure 1). Ferroptosis is defined as an iron-dependent cell death modality driven by glutathione peroxidase 4 (GPX4) inactivation and subsequent lipid peroxidation of polyunsaturated fatty acids (PUFAs) in cellular membranes (Peng et al., 2022). It has been increasingly linked to therapy resistance and metastatic progression (Liu X. et al., 2022) as well as emerging therapeutic strategies, including those derived from traditional Chinese medicine (Qin et al., 2024; Mi et al., 2025; Yang et al., 2024; Peng et al., 2024). Anoikis represents an integrin-dependent apoptotic process, triggered when cells lost adhesion to the extracellular matrix, thereby activating mitochondrial pathways and caspase cascades (Taddei et al., 2012). Pyroptosis is characterized as an inflammatory cell death pathway, mediated by inflammasome-dependent activation of caspase-1 or caspase-4/5/11, which cleave gasdermin proteins to form membrane pores, resulting in osmotic lysis and release of pro-inflammatory factors (Peng et al., 2022). Recent transcriptomic studies further show that pyroptosis-related gene signatures can predict prognosis and reveal regulatory axes (Zhou et al., 2022).

MicroRNA (miRNA) emerged as key regulators of RCDs by modulating gene expression, signaling pathways, and metabolic homeostasis. MiRNAs are small single-stranded RNA molecules that play a negative role in gene expression through interactions with mRNA. They are produced from hairpin precursors by Drosha and Dicer, usually comprising approximately 22 nucleotides (Mohr and Mott, 2015). Substantial evidence indicated that miRNAs functioned either as oncogenic drivers or tumor suppressors in cell death pathways, profoundly shaping tumorigenesis and therapeutic responses (Chen M. et al., 2025; Di Leva et al., 2014; Zhang et al., 2023; Fang et al., 2025). Recent studies have further expanded this understanding by revealing complex regulatory layers. For instance, the m6A modification of miRNAs has been shown to influence their processing and stability, impacting cancer progression (Mehmood, 2024). Moreover, natural compounds, such as resveratrol, can exert anticancer effects by modulating miRNA expression networks (Keshavarzmotamed et al., 2024; Wang J. Y. et al., 2025). In specific cancer types, the interplay between miRNAs and key signaling pathways has also been elucidated; for example, dysregulated miRNAs targeting the p53 signaling pathway have been identified in triple-negative breast cancer (Naeimzadeh et al., 2024). Additionally, the renin-angiotensin-aldosterone system has emerged as a novel axis through which miRNAs influence cancer biology, particularly in the context of chemoradiotherapy resistance (Li et al., 2025).

Given the central role of miRNAs in RCD regulation, a key issue is to delineate how different miRNAs mechanistically modulate specific RCD pathways during cancer progression. MiRNAs interact within elaborate regulatory networks, regulating the core components and sensors of different RCD forms. A thorough analysis of these mechanisms is crucial for clarifying their roles in tumor progression and treatment. Therefore, this review focuses on the regulatory involvement of miRNAs in three RCDs, while establishing a theoretical foundation for innovative antitumor approaches and pointing to novel avenues for biomarker discovery.

## 2 Ferroptosis

### 2.1 A brief overview of ferroptosis

Ferroptosis is a new form of oxidative and non-apoptotic RCD dependent on iron ions and lipid peroxidation, with significant tumor-suppressive potential (Jiang et al., 2021; Lei et al., 2024). Mechanistically, intracellular PUFAs are esterified and incorporated into membrane phospholipids by acyl-CoA synthetase long-chain family member 4 (ACSL4) and lysophosphatidylcholine acyltransferase 3 (LPCAT3) (Doll et al., 2017; Reed et al., 2022), generating PUFA-containing phospholipids (PUFA-PLs). The subsequent accumulation of lipid hydroperoxides (PUFA-PL-OOH) progressively disrupts membrane integrity and ultimately induces ferroptosis. This process is promoted by free ferrous iron (Fe^2+^), which reacts with hydrogen peroxide via the Fenton reaction to produce hydroxyl radicals (·OH) that attack PUFA-PLs and yield PUFA-PL-OOH (Liang et al., 2022). Conversely, the antioxidant defense system, particularly the System Xc^−^/GSH/GPX4 axis, normally clears these lipid hydroperoxides. However, when the system is impaired, depletion of glutathione (GSH) and inactivation of GPX4 prevent the clearance of lipid hydroperoxides (Kuang et al., 2020), exacerbating membrane disruption and driving ferroptosis.

### 2.2 Interaction between ferroptosis and MiRNAs in cancers

**FIGURE 2:**
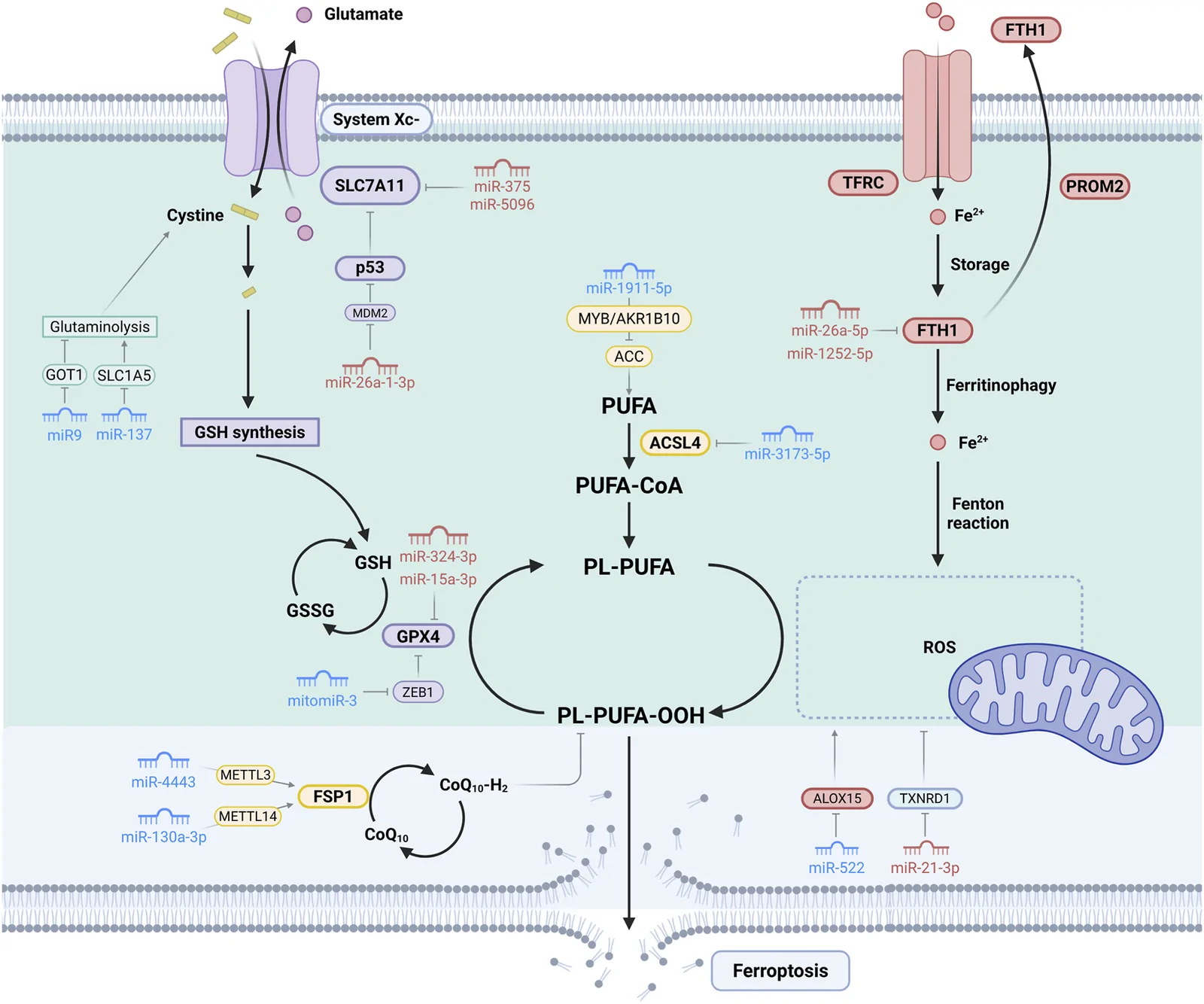
Regulation mediated by miRNAs in ferroptosis. This schematic summarizes the major miRNAs reported to modulate ferroptosis by targeting key components of canonical (green background) and non-canonical (blue background) pathways. Red-labeled miRNAs denote positive regulators that enhance ferroptosis susceptibility, whereas blue-labeled miRNAs indicate suppressors that inhibit ferroptosis. A substantial proportion of identified miRNAs converge on the key ferroptosis regulators, particularly SLC7A11 and GPX4, which influence cystine import, GSH metabolism, and lipid peroxide detoxification. Additional miRNAs act through pathways controlling lipid peroxidation (e.g., ROS production, ACSL4-dependent PUFA activation) and iron metabolism (e.g., regulation of TFRC and related iron-handling proteins).

Increasing evidence demonstrated that miRNAs played vital roles in regulating ferroptosis (Zhang and Xie, 2024; Zuo et al., 2022). Multiple classes of miRNAs, modulated antioxidant defense, iron metabolism, and lipid peroxidation, thereby exerting fine control over the initiation and progression of ferroptosis (Table 1) (Figure 2). Exploration of the interaction between miRNAs and ferroptosis provided critical insights into the molecular basis of cancer cell’s RCD and offered new opportunities for biomarker discovery and therapeutic intervention.

**TABLE 1:** MiRNAs modulating ferroptosis in cancers.

MiRNA name	Cancer	Target/regulatory axis	Effect on ferroptosis	References
miR-15a-3p	Colorectal cancer	GPX4	Promote	Liu L. et al. (2022)
miR-130a-3p	Endometrial carcinoma	METTL14/FSP1	Inhibit	Han et al. (2025)
miR-375	Gastric cancer	SLC7A11	Promote	Ni et al. (2021)
miR-522	Gastric cancer	ALOX15	Inhibit	Zhang H. et al. (2020)
miR-1291	Gastric cancer	FOXA2/GPX4	Promote	Qian et al. (2025)
miR-1911-5p	Gastric cancer	MYB/ACC AKR1B10/ACC	Inhibit	Kong et al. (2025)
miR-21-5p	Hepatocellular carcinoma	MELK/AKT/mTOR	Inhibit	Hu et al. (2023)
miR-9	Melanoma	GOT1	Inhibit	Zhang et al. (2018)
miR-21-3p	Melanoma	TXNRD1	Promote	Guo et al. (2022)
miR-137	Melanoma	SLC1A5	Inhibit	Luo et al. (2018)
miR-26a-1-3p	Non-small cell lung cancer	MDM2/p53	Promote	Fu et al. (2024)
miR-139	Non-small cell lung cancer	c-JUN and KPNA2/NrF2	Promote	Zhang L. et al. (2024)
miR-4443	Non-small cell lung cancer	METTL3/FSP1	Inhibit	Song et al. (2021)
miR-26a-5p	Osteosarcoma	FTH1	Promote	He et al. (2024)
miR-144-3p	Osteosarcoma	ZEB1/GPX4	Promote	Jiang et al. (2023)
miR-3173-5p	Pancreatic ductal adenocarcinoma	ACSL4	Inhibit	Qi et al. (2023)
miR-181a-5p	Prostate cancer	LATS1/Hippo/YAP1	Inhibit	Xiao et al. (2024)
miR-1252-5p	Prostate cancer	FTH1	Promote	Lu et al. (2024)
miR-324-3p	Renal cell carcinoma	GPX4	Promote	Yu et al. (2022)
miR-5096	Triple-negative breast cancer	SLC7A11	Promote	Yadav et al. (2021)
mitomiR-3	Triple-negative breast cancer	ZEB1/GPX4	Inhibit	Kandettu et al. (2025)

#### 2.2.1 MiRNAs modulate ferroptosis in a SLC7A11-dependent manner

System Xc^−^ imports cystine in exchange for glutamate. This cystine fuels GSH synthesis, which suppresses lipid peroxidation. As the core subunit of this antiporter, solute carrier family 7 member 11 (SLC7A11) drives cystine uptake and dictates ferroptosis susceptibility. Its expression is tightly controlled by multiple miRNA species. By governing SLC7A11 abundance, these miRNAs directly shape cystine import capacity, GSH biosynthesis, and ultimately the threshold for ferroptosis induction.

MiRNAs can directly target SLC7A11 in cancer cells. miR-375 was reported to attenuate the stemness of gastric cancer (GC) cells both *in vitro* and *in vivo* by downregulating SLC7A11 to trigger ferroptosis, thereby slowed the GC progression (Ni et al., 2021). In breast cancer (BC), miR-5096 inhibited the tumorigenesis through direct targeting on SLC7A11 to promote ferroptosis. Moreover, miR-5096 also regulated the epithelial-mesenchymal transition (EMT) process in a tumor xenograft model of zebrafish larvae (Yadav et al., 2021). Beyond these, miRNAs can regulate ferroptosis via upstream factors such as p53 and FOXA2. p53 is a well-established tumor suppressor. It functions primarily as a transcription factor to regulate genes involved in cell cycle arrest, apoptosis, and metabolism. p53 has been shown to repress SLC7A11 expression. In non-small cell lung cancer (NSCLC), miR-26a-1-3p targeted and downregulated an E3 ubiquitin ligase MDM2, which promoted p53 activity and consequently decreased expression of SLC7A11 (Fu et al., 2024). Given that SLC7A11 is one of the most extensively studied targets in ferroptosis regulation, these miRNAs hold significant promise as druggable targets for cancer therapy.

#### 2.2.2 MiRNAs modulate ferroptosis in a GPX4-dependent manner

GPX4 is a selenium-containing enzyme that serves as the master suppressor of ferroptosis. As an essential phospholipid peroxidase, GPX4 utilized GSH to neutralize lipid peroxides, directly preventing membrane damage. Thus, GPX4 activity constituted the final barrier against ferroptosis.

MiRNAs also function as important regulators of GPX4. They can inhibit ferroptosis by suppressing ZEB1, subsequently increasing GPX4 expression. In basal-like TNBC cells, the overexpression of mitochondrial genome–encoded mitomiR-3 inhibited ZEB1 expression and inhibited EMT. In mitomiR-3–sponge cells, ZEB1 upregulation directly suppressed GPX4 expression, accompanied by elevated intracellular levels of PUFA metabolites, increased Fe^2+^ content, and enhanced lipid ROS following mitomiR-3 inhibition (Kandettu et al., 2025). Conversely, in osteosarcoma (OS), miR-144-3p directly repressed ZEB1, resulting in increased cytosolic Fe^2+^ concentration and induced lipid ROS accumulation *in vitro* and *in vivo*, contributing to ferroptosis occurrence and repressing tumor progression (Jiang et al., 2023). MiRNAs can also promote ferroptosis by directly downregulating GPX4. In renal cell carcinoma, miR-324-3p directly targeted and downregulates GPX4, independent of p53, and subsequently promoted ferroptosis (Yu et al., 2022). Besides, miR-15a-3p could bind to the 3′-untranslated region of GPX4 and further reduced the level of GPX4, contributing to accumulation of ROS, Fe^2+^, and malondialdehyde (MDA) in colorectal cancer cells (Liu L. et al., 2022). In GC, miR-1291 levels were increased to suppress FOXA2. FOXA2 downregulation led to the increase of ferritin expression, alongside decrease in GPX4. The promoting effect of miR-1291 was also confirmed in further *in vivo* studies (Qian et al., 2025). The direct engagement underscores its potential as a specific and effective therapeutic candidate for colorectal cancer.

#### 2.2.3 MiRNAs modulate ferroptosis in an iron metabolism-dependent manner

Iron metabolism played a pivotal role in ferroptosis, as intracellular iron availability determined the extent of lipid peroxidation. Increased uptake through transferrin receptor (TFRC), reduced storage by ferritin heavy chain 1 (FTH1), or ferritin degradation via NCOA4 expanded the labile iron pool and accelerated ferroptosis.

MiRNAs primarily regulated iron metabolism via FTH1 in ferroptosis. In OS, METTL1 stabilized FTH1 mRNA and pri-miR-26a through m^7^G methylation. miR-26a-5p, in turn, inhibited FTH1 translation, resulting in elevated FTH1 mRNA levels alongside reduced protein abundance. The reduction in FTH1 protein increased cytosolic Fe^2+^, driving ferroptosis (He et al., 2024). In prostate cancer, a pseudogene FTH1P8 suppressed ferroptosis by enhancing FTH1 expression via miR-1252-5p (Lu et al., 2024). Therefore, miRNA-mediated post-transcriptional regulation of FTH1 is a critical determinant of intracellular iron availability and ferroptosis susceptibility in cancer.

#### 2.2.4 MiRNAs modulate ferroptosis in an ACSL4-dependent manner

ACSL4 is established as a key determinant of ferroptosis. By incorporating PUFAs into membrane phospholipids, ACSL4 enable lipid peroxidation and further drove ferroptosis. Given its central role in defining cellular sensibility to ferroptosis, ACSL4-associated pathways receive substantial attention. However, only a few studies have explored the roles of miRNAs in regulating ACSL4, most of which have focused on the competing endogenous RNA mechanism involving circular RNAs or long non-coding RNAs. To date, only one study has reported a direct inhibitory effect of a miRNA on ferroptosis. Pancreatic ductal adenocarcinoma cell lines co-cultured with cancer-associated fibroblasts developed gemcitabine resistance after chemotherapy. Chemotherapy upregulated miR-3173-5p, which repressed ACSL4 expression, thereby attenuating gemcitabine-induced ferroptosis. *In vivo* studies confirmed the suppressive effect of miR-3173-5p on ferroptosis (Qi et al., 2023). Future investigations should focus on the regulatory logic underlying ACSL4 post-translational modifications and its functional interplay with other ACSL family members, with the goal of achieving precise modulation of ferroptosis sensitivity across diverse tumor types by targeting this critical node.

#### 2.2.5 MiRNAs modulate ferroptosis in other manners

##### 2.2.5.1 MiRNAs regulating ferroptosis via the classical signaling pathways

The PI3K/AKT/mTOR pathway served as a central metabolic hub linking growth signals, nutrients, and redox balance, and its dysregulation influenced ferroptosis by altering ROS, lipid metabolism, and iron homeostasis. MiRNAs regulate ferroptosis by directly modulating the PI3K/AKT/mTOR axis. In hepatocellular carcinoma (HCC), miR-21-5p upregulated maternal embryonic leucine zipper kinase (MELK), which enhanced the cellular antioxidant defense, suppressed lipid peroxidation, and limited iron ion accumulation via activation of the AKT/mTOR axis (Hu et al., 2023).

Ferroptosis suppressor protein 1 (FSP1), also known as AIFM2, functions as a potent ferroptosis inhibitor by reducing coenzyme Q10 (CoQ10) to its lipophilic antioxidant form, preventing lipid peroxidation independently of the GPX4 pathway. Several miRNAs regulate ferroptosis by targeting m6A regulators of FSP1 and stabilizing its expression. In EC, cisplatin-resistant cells and their exosomes (CisR-exos) display higher levels of miR-130a-3p relative to parental cells and CisS-exos. miR-130a-3p bound directly to METTL14, attenuating m6A methylation of FSP1 transcripts. This reduction limited FSP1 degradation and stabilized its expression to suppress ferroptosis (Han et al., 2025). Similarly, in NSCLC, miR-4443 was demonstrated to control the efficacy of chemotherapeutic regimen. miR-4443 could directly bind to gene METTL3 in an m6A manner and further rescued the cisplatin-induced downregulation of FSP1 (Song et al., 2021).

NrF2 is a central transcriptional regulator of cellular redox homeostasis. Under stress conditions, it drives the expression of antioxidant and iron-handling genes, including SLC7A11, GCLC, GPX4, and HO-1, to limit lipid peroxidation and restrain ferroptosis. When NrF2 is destabilized or its nuclear import is impaired, these defenses collapse, making cells more vulnerable to ferroptotic damage (Zheng et al., 2025). Specific miRNAs attenuate NrF2 activity by repressing factors required for its transcriptional activation or nuclear import, ultimately constraining the expression of NrF2-dependent antioxidant genes. In NSCLC, miR-139 suppressed NrF2 signaling by inhibiting c-JUN and KPNA2, and the latter facilitating NrF2 nuclear import. NrF2 downregulation reduced the expression of pivotal downstream genes, including SLC7A11, GCLC, GPX4 and HO-1, leading to elevated intracellular MDA and lipid ROS. Further study showed that ionizing radiation (IR) increased EGR1 expression, which upregulated the transcription activity of miR-139 (Zhang L. et al., 2024).

The Hippo signaling pathway regulates yes associated protein (YAP) and transcriptional coactivator with PDZ-binding motif (TAZ) to modulate the expression of ferroptosis-related genes involved in iron uptake and lipid metabolism, while miRNAs inhibited ferroptosis by targeting Hippo pathway components. In prostate cancer (PCa), taurine activated the LXRα/SCD1 axis to upregulate miR-181a-5p and its partner FUS, promoting its enrichment in extracellular vesicles delivered to tumor-associated macrophages (TAMs). In TAMs, miR-181a-5p repressed LATS1, inhibited Hippo signaling, and activated YAP1-driven transcription, reinforcing M2 polarization and taurine uptake, which ultimately suppressed ferroptosis (Xiao et al., 2024). This interplay illustrates how miRNA-mediated crosstalk between tumor cells and macrophages links iron metabolism to immune polarization, positioning ferroptosis resistance as an integrated outcome of both cell-autonomous and microenvironmental reprogramming.

##### 2.2.5.2 MiRNAs regulating ferroptosis through ROS metabolism

Ferroptosis arises from the breakdown of the balance between lipid peroxidation and antioxidant defenses, which is primarily driven by disrupted ROS metabolism. Iron-dependent Fenton reactions generate excess hydroxyl radicals that attack polyunsaturated fatty acids and initiate a lethal lipid-peroxidation cascade, while the GSH–GPX4 axis removes peroxides to maintain redox homeostasis.

Many miRNAs exert inhibitory effects on ferroptosis by targeting key molecules involved in ROS production and reducing lipid peroxidation. In GC, exosome-miR-522 was derived from cancer-associated fibroblasts (CAFs) in the tumor microenvironment and could directly bind to arachidonate lipoxygenase 15 (ALOX15) which functioned as a lipid-ROS generator. miR-522 was packed into exosomes by heterogeneous nuclear ribonucleoprotein A1 (hnRNPA1) whereas the ubiquitin-specific protease 7 (USP7) stabilized hnRNPA1 through de-ubiquitination. Cisplatin and paclitaxel were found to increase the secretion of miR-522 from CAFs and decrease lipid-ROS to lower the chemo-sensitivity via USP7/hnRNPA1 axis (Zhang H. et al., 2020). miR-1911-5p downregulated MYB and upregulated AKR1B10 to inhibit ACC-pathway–mediated ferroptosis. Exosomal miR-1911-5p, transferred from TAMs to GC cells, contributed to cisplatin resistance by suppressing ferroptosis (Kong et al., 2025).

In contrast, some miRNAs promote ferroptosis by driving ROS accumulation. In melanoma cells, ATF3-induced upregulation of miR-21-3p enhanced IFN-γ–induced ferroptosis by attenuating the suppressive activity of TXNRD1 on lipid ROS accumulation (Guo et al., 2022).

Beyond antioxidant defenses and lipid peroxidation, miRNAs inhibit ferroptosis through glutaminolysis-dependent redox control. In melanoma cells, as an important regulator of ferroptosis, miR-9 enabled to inhibit erastin- and RSL3-induced ferroptosis in melanoma cells. It directly targeted GOT1 by binding to its 3′-UTR. Moreover, miR-9 deficiency mediated lipid ROS accumulation and ferroptosis could be abrogated by inhibiting glutaminolysis processs (Zhang et al., 2018). Apart from this, miR-137 decreased the sensitivity of melanoma cells to erastin- and RSL3-indcued ferroptosis in melanoma both *in vitro* and *in vivo*. Meanwhile, miR-137 knockdown reversed this effect and enhanced ferroptosis. Mechanistically, miR-137 physically bound to SLC1A5 which was a glutamine transporter and inhibited glutaminolysis to regulate the redox homeostasis (Luo et al., 2018).

Taken together, these studies show that miRNAs regulate ferroptosis not only via canonical antioxidant pathways but also through direct control of ROS metabolism, encompassing antioxidant defenses, ROS generation, lipid peroxidation, and glutaminolysis-driven redox balance.

Collectively, these findings highlight that miRNAs regulate ferroptosis not only through classical pathways such as SLC7A11, GPX4, iron metabolism or ACSL4, but also via diverse non-canonical mechanisms. The latter primarily involve indirect modulation of signaling cascades (e.g., PI3K/AKT/mTOR, Nrf2, and Hippo) as well as processes related to ROS metabolism. This multilayered regulation underscores the complexity of miRNA networks in ferroptosis and their potential as therapeutic targets across malignancies.

## 3 Anoikis

### 3.1 A brief overview of anoikis

Anoikis, a term derived from the Greek word for “homelessness”, was a distinct form of programmed cell death initiated when anchorage-dependent cells detached from the extracellular matrix and lost appropriate cell-matrix interactions (Wang Y. et al., 2024; Paoli et al., 2013). In normal tissues, this process functioned as a critical homeostatic mechanism and a fundamental barrier against tumorigenesis by eliminating displaced cells and preventing their colonization of ectopic sites. Consequently, the ability to evade anoikis was a hallmark of metastatic cancer cells, enabling their survival during transit in the circulatory or lymphatic systems and subsequent growth at distant locations (Wang Y. et al., 2024; Nepali and Kyprianou, 2023).

**FIGURE 3:**
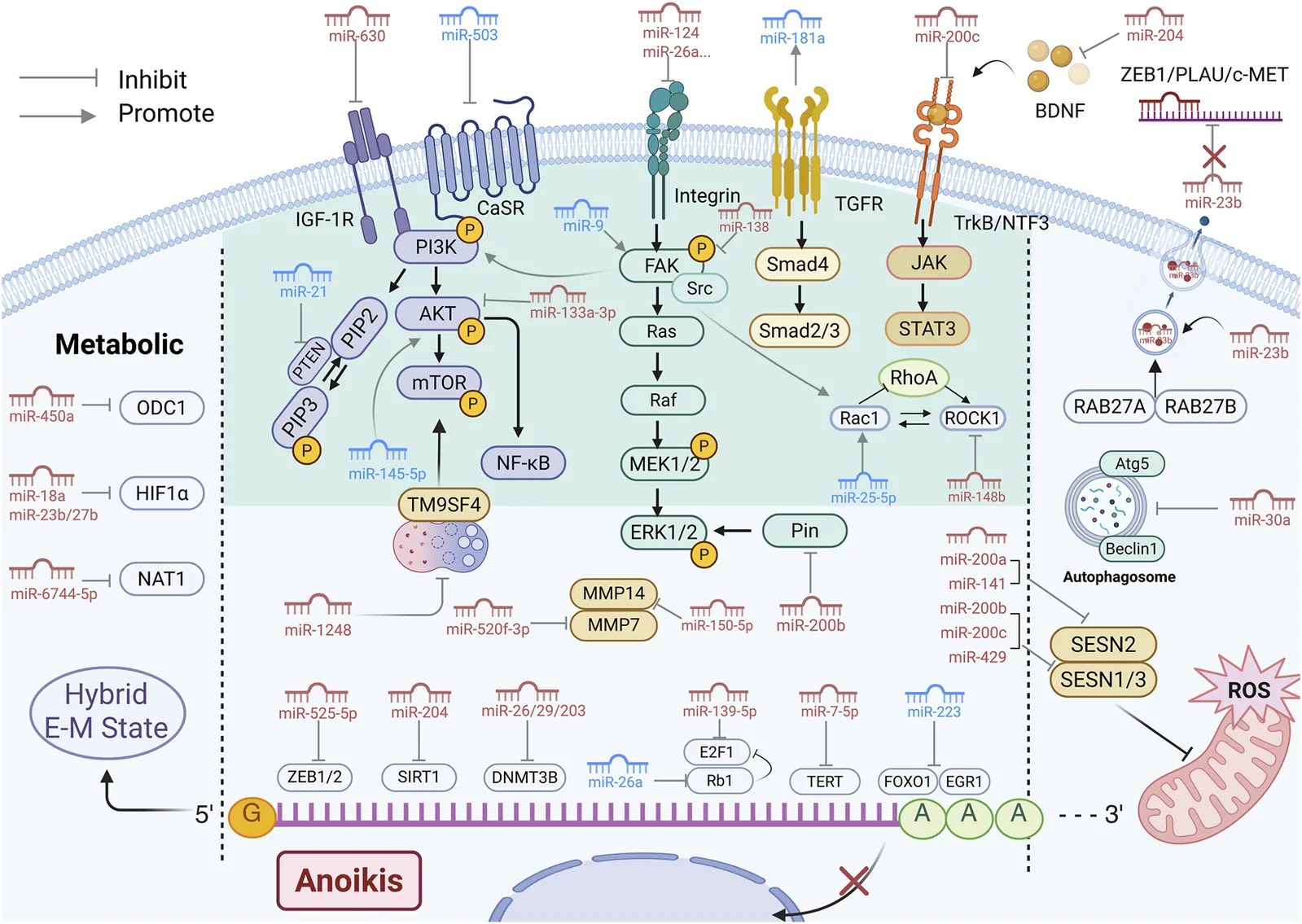
The multifaceted landscape of MiRNA-mediated regulation in the anoikis-resistance cascade. This schematic delineates the sophisticated regulatory network of miRNAs that ensures tumor cell survival and promotes metastatic dissemination upon detachment from the ECM. On the left, the diagram highlights miRNA-driven metabolic reprogramming, where specific miRNAs modulate lipid metabolism to sustain bioenergetic homeostasis under stress. Centrally, the orchestration of core survival signaling is illustrated: miRNAs target membrane receptors to activate downstream PI3K–AKT, MAPK–ERK, and TGFR cascades (with canonical pathways denoted by a dark green background), thereby bypassing apoptotic triggers. On the right, the focus shifts to the maintenance of cellular integrity, featuring miRNA regulation of mitochondrial ROS scavenging, autophagy. On the upper-right, cancer cells selectively export the tumor-suppressive miR-23b cluster, and this intracellular depletion relieves the repression on target mRNAs. This de-repression leads to their significant upregulation, driving a hybrid E-M state and metastatic properties. At the bottom, the schematic depicts the targeting of mRNAs encoding key effectors in the cytoplasm. The miRNA-mediated depletion of these proteins subsequently blocks their translocation into the nucleus, preventing them from exerting their nuclear functions. Representative miRNAs are explicitly labeled at their respective functional nodes. In this updated schematic, solid T-bars strictly represent canonical miRNA-mediated translational repression or mRNA degradation occurring in the cytoplasm. Arrows indicate downstream biological processes, signaling activation, or translocation dynamics. Red crosses (“×”) denote the abrogation of canonical inhibition or the blockade of protein nuclear entry.

The execution of anoikis was primarily orchestrated through the canonical intrinsic and extrinsic apoptotic pathways, which both converged on the activation of effector caspases. The intrinsic (or mitochondrial) pathway acted as a central axis in this process. Upon cell detachment, the loss of integrin-mediated survival signals led to the activation and release of pro-apoptotic BH3-only proteins, most notably Bim, from cytoskeletal complexes. Bim then neutralized anti-apoptotic Bcl-2 family members (e.g., Bcl-2, Bcl-xL), thereby unleashing the pro-apoptotic effector proteins Bax and Bak (Paoli et al., 2013). These effectors oligomerized on the mitochondrial outer membrane, inducing mitochondrial outer membrane permeabilization (MOMP) and the release of Cytochrome C (Cyt C). Cytosolic Cyt C subsequently bound to Apaf-1 and pro-caspase-9 to form the apoptosome, initiating a caspase cascade that culminated in cell death. Additionally, the extrinsic pathway, triggered by the ligation of death receptors (e.g., Fas or TRAIL), contributed to anoikis by activating caspase-8. Active caspase-8 could directly cleave effector caspases or engage the intrinsic pathway via the cleavage of Bid (tBid) to amplify the apoptotic signal (Han et al., 2023; Figure 3).

Cancer cells, however, have developed numerous strategies to develop anoikis resistance, a critical step for metastatic progression. These mechanisms include: (i) Activation of oncogenic pathways, where hyperactivation of pro-survival signaling cascades like PI3K/AKT/mTOR, RAS/MAPK, and NF-κB provides potent anti-apoptotic signals that override death stimuli (Paoli et al., 2013); (ii)Alterations in cell adhesion signaling, such as switching integrin expression profiles to adapt to new matrix environments or overexpressing focal adhesion kinase (FAK) to sustain survival signals (Wang Y. et al., 2024); (iii) Metabolic reprogramming, including shifts in glucose, lipid, and amino acid metabolism, which helps detached cells cope with oxidative stress and energy demands (Wang Y. et al., 2024; Raeisi et al., 2022); (iv) Induction of EMT, a developmental program that endows cancer cells with migratory capabilities and inherent resistance to anoikis (Nepali and Kyprianou, 2023); and (v) Influence of the tumor microenvironment (TME), where factors like altered matrix stiffness and stromal-derived growth factors or cytokines create a protective niche that supports the survival of detached cells (Nepali and Kyprianou, 2023). This multifaceted resistance allows cancer cells to survive the perilous journey through the circulation and successfully establish distant metastases.

### 3.2 Interaction between anoikis and MiRNAs in cancers

Consequently, targeting anoikis required dissecting a redundant and highly plastic regulatory web. It was within this intricate landscape that miRNAs emerged as pivotal players. Acting as master regulators, miRNAs could simultaneously modulate multiple nodes of this signaling network (错误!书签自引用无效。) (Table 2). This part focused on unraveling this complexity, with a specific emphasis on how diverse miRNAs served as critical switches within this molecular landscape, offering novel pharmacological entry points to dismantle the metastatic state and overcome anoikis resistance. To systematically delineate the multifaceted roles of miRNAs in conferring anoikis resistance, we categorized the underlying mechanisms into a three-stage “Anoikis Escape” paradigm. Established upon the spatiotemporal progression of the anoikis cascade, the framework was comprised of the following three phases (Fu et al., 2022): Remodeling the Detached State and Mimicking Anchorage, wherein malignant cells effectively abrogated the initiation of death signals at the membrane source by simulating a pseudo-anchored phenotype (D’Arcy, 2019); Rewiring Survival Signaling and Metabolic Adaptation: a compensatory phase involved the autonomous reprogramming of intracellular kinase networks and metabolic flux to sustain bioenergetics under suspension stress (Fulda, 2009); Direct Blockade of the Apoptotic Machinery: which functioned as the ultimate fail-safe mechanism to inhibit mitochondrial executioners and prevent the terminal realization of apoptosis. The schematic representation provides an integrative overview of the regulatory network, with representative miRNAs and their functional targets explicitly highlighted (Figure 3).

**TABLE 2:** MiRNAs modulating anoikis in cancers.

MiRNA name	Cancer	Target/regulatory axis	Effect on anoikis	References
miR-23b	Bladder cancer	FSCN1, PLAU, ZEB1/2	Inhibit	Ostenfeld et al. (2014)
miR-124	Breast cancer	IQGAP1, LAMB1, ITGB1	Promote	Lv et al. (2011)
miR-148b	Breast cancer	ROCK1	Promote	Cimino et al. (2013)
miR-181a	Breast cancer	Bim	Inhibit	Taylor et al. (2013)
miR-181a	Breast cancer	TGF-β	Inhibit	Polo-Generelo et al. (2022)
miR-18a	Breast cancer	HIF1A	Inhibit	Krutilina et al. (2014)
miR-200a	Breast cancer	YAP1	Inhibit	Yu et al. (2013)
miR-200b	Breast cancer	Pin1	Promote	Zhang X. et al. (2013)
miR-200c	Breast cancer	MSN, FN1	Promote	Howe et al. (2011)
miR-200c	Breast cancer	TrkB/NTF3	Promote	Howe et al. (2012)
miR-223	Breast cancer	FOXO1	Inhibit	Pinatel et al. (2014)
miR-630	Breast cancer	IGF1R	Promote	Corcoran et al. (2014)
miR-6744-5p	Breast cancer	NAT1	Promote	Malagobadan et al. (2020)
miR-525-5p	Cervical cancer	UBE2C/ZEB1/2 axis	Promote	Chen and Liu (2020)
miRNA (GRSF1)	Cervical cancer	PIK3R3/AKT/NF-κB	Inhibit	Sun et al. (2019)
miR-520f-3p	Chondrosarcoma	MMP7	Promote	Nguyen et al. (2023)
miR-10a	Colorectal cancer	KLF4	Promote	Liu et al. (2017b)
miR-124	Colorectal cancer	ITGA3	Promote	Sa et al. (2018)
miR-145-5p	Colorectal cancer	PI3K/AKT	Inhibit	Cheng et al. (2022)
miR-503	Colorectal cancer	CaSR	Inhibit	Noguchi et al. (2016)
miR-200 family	Endometrial cancer	Sestrin1/2/3	Promote	Kozak et al. (2019)
miR-145	Esophageal Adeno	ITGA5	Inhibit	Derouet et al. (2014)
miR-21	Esophageal Adeno	PTEN, PDCD4	Inhibit	Zhao et al. (2018)
miR-26a	Esophageal Adeno	Rb1-E2F1	Inhibit	Zhang Y. F. et al. (2013)
miR-138	Ewing’s sarcoma	FAK	Promote	Tanaka et al. (2016)
miR-204	Gastric cancer	SIRT1	Promote	Zhang L. et al. (2013)
miR-497	Gastric cancer	Wnt/β-catenin	Promote	Yu et al. (2023)
miR-30b/30c	General/screen	Caspase-3	Inhibit	Moreno-Mateos et al. (2013)
miR-25-5p	HCC	RhoGDI	Inhibit	Liu et al. (2018)
miR-26a	HCC	ITGA5	Promote	Zhang et al. (2015)
miR-30a	HCC	Beclin 1/Atg5	Promote	Fu et al. (2018)
miR-424-5p	HCC	ICAT	Promote	Zhang et al. (2014)
miR-7/21/107	HCC	Maspin	Inhibit	Chen et al. (2015)
miR-9	HCC	Integrin β1/FAK	Inhibit	Liu et al. (2017a)
miR-1827	Lung adenocarcinoma	CAV1	Promote	Guo et al. (2020)
miR-150-5p	Lung cancer	ERK/MMP-14	Promote	Thuong et al. (2024)
miR-451	Lung cancer	MIF	Promote	Wang et al. (2011)
miR-138-5p	Melanoma	p53	Inhibit	da Cruz et al. (2021)
miR-214	Melanoma	TFAP2, ALCAM	Inhibit	Penna et al. (2013)
miR-214	Melanoma	TFAP2C	Inhibit	Penna et al. (2011)
miR-26b/203	Melanoma	DNMT3B	Promote	Gasque Schoof et al. (2015)
miR-29b/c	Melanoma	DNMT3A/DNMT3B	Promote	Gasque Schoof et al. (2015)
miR-141	Ovarian cancer	Survivin (via KLF12)	Inhibit	Mak et al. (2017)
miR-200c	Ovarian cancer	ZEB1/2	Promote	Cittelly et al. (2012)
miR-204	Ovarian cancer	BDNF	Promote	Yan et al. (2015)
miR-450a	Ovarian cancer	ODC1	Promote	Muys et al. (2019)
miR-222-3p	Pancreatic cancer	PUMA	Inhibit	Wei et al. (2017)
miR-363-3p	Papillary thyroid	ITGA6	Promote	Pan et al. (2019)
miR-99a-3p	Papillary thyroid	GRP94	Promote	Gao et al. (2021)
miR-1247	Prostate cancer	Stromal signals	Promote	Taddei et al. (2019)
miR-132	Prostate cancer	Methylation	Promote	Formosa et al. (2013)
miR-133a-3p	Prostate cancer	PI3K/AKT	Promote	Tang et al. (2018)
miR-23b/-27b	Prostate cancer	Fascin-, HIP1R	Promote	Rice et al. (2016), Ishteiwy et al. (2012)
miR-203	Various carcinomas	DKK1, Wnt/β-catenin	Promote	Taube et al. (2013)
KSHV-miRNAs	Viral cancers	TPM1	Inhibit	Kieffer-Kwon et al. (2015)

#### 3.2.1 Remodeling the detached state and mimicking anchorage

Under physiological conditions, the disruption of interactions between the cell and the ECM served as the primary trigger for anoikis. Malignant cells, however, orchestrated a “deceptive” strategy during this initiation phase. Research demonstrated that miRNAs modulated the cytoskeleton, adhesion receptors, and cellular morphology to simulate a pseudo-anchored state or to render the cells independent of anchorage signals, thereby preventing the generation of the death signal at its source (Fu et al., 2022): EMT: miRNAs regulated core transcription factors (e.g., ZEB1/2, Snail, Twist, KLF4) and altered the balance between E-cadherin and N-cadherin, endowing cells with the plasticity required to survive suspension (D’Arcy, 2019); Integrin Switching: Aberrant expression of specific Integrin subunits (ITGA/ITGB) was induced to maintain pro-survival signaling despite the loss of native ECM attachment (Fulda, 2009); Cytoskeletal Remodeling: miRNAs targeted structural proteins such as Vimentin and Fascin, as well as regulators like Rho GTPases, to reinforce cellular rigidity and prevent structural collapse (Hanahan and Weinberg, 2011); Focal Adhesion Signaling: The modulation of FAK (PTK2) and Src kinases was identified as a critical step in converting mechanical detachment into intracellular survival cues.

Investigations into aggressive BC cell lines revealed that the restoration of miR-200c (Howe et al., 2011) significantly inhibited cell motility and overcame anoikis resistance. Mechanistically, miR-200c functioned as a master regulator by concurrently repressing a broad network of mesenchymal genes, including Moesin (MSN) and Fibronectin (FN1). Complementing this, miR-148b (Cimino et al., 2013) was identified as a major coordinator of progression by targeting ROCK1, preventing cytoskeletal reorganization. Furthermore, miR-124 (Lv et al., 2011) was shown to suppress multiple steps of metastasis by simultaneously targeting a cohort of pro-metastatic genes, including IQGAP1, LAMB1, and ITGB1, effectively dismantling the cell’s adhesive capabilities.

In the context of HCC, the downregulation of specific tumor-suppressive miRNAs plays a pivotal role in initiating metastasis. miR-26a (Zhang et al., 2015) was identified to exert a potent tumor-suppressive function by directly targeting the 3′-UTR of Integrin α5 (ITGA5). The consequent downregulation of ITGA5 protein levels disrupted the primary fibronectin receptor signaling complex on the cell surface, which in turn inactivated the downstream Akt survival pathway, rendering cells vulnerable to detachment. Furthermore, miR-424-5p (Zhang et al., 2014) was found to critically reverse the EMT program. By directly targeting ICAT, a negative regulator of the Wnt pathway, this miRNA stabilized the E-cadherin /β− catenin complex at the plasma membrane, thereby reinforcing cell-cell adhesion and sensitizing HCC cells to anoikis. Conversely, epigenetic dysregulation also contributes to this phase; aberrant Fibronectin glycosylation resulting from the loss of the glycosyltransferase GALNT4 was shown to transcriptionally upregulate miR-9 (Liu et al., 2017a). High levels of miR-9 subsequently hyperactivated the Integrin β1/ FAK signaling axis, providing a sustained, albeit aberrant, survival signal.

For GC, miR-204 (Zhang L. et al., 2013) was highlighted as a crucial regulator of cellular plasticity that targeted SIRT1. The inhibition of the SIRT1-LKB1 axis by this miRNA resulted in the upregulation of epithelial markers like E-cadherin and the downregulation of mesenchymal markers such as Vimentin, effectively reverting the EMT phenotype. Additionally, miR-497 (Yu et al., 2023) functioned as a critical suppressor of the mesenchymal phenotype in this malignancy. *In vivo* experiments demonstrated that its restoration significantly upregulated epithelial markers while dismantling structural plasticity via the blockade of Wnt/ β -catenin signaling, thereby inhibiting tumor growth and metastasis. In esophageal adenocarcinoma, miR-145 (Derouet et al., 2014) expression was found to accelerate progression by modulating extracellular matrix components and adhesion-related genes, enhancing invasive capacity.

Regarding colorectal malignancies, miR-10a (Liu et al., 2017b) exerted its suppressive effect by targeting the 3′-UTR of KLF4. This targeting event led to the downstream downregulation of FN1, a key ECM component, resulting in the reversal of EMT and increased sensitivity to detachment. Similarly, miR-124 (Sa et al., 2018) was found to directly target Integrin α3 (ITGA3) in CRC cells. The suppression of ITGA3 reduced the anchorage signals derived from the matrix, forcing cells into apoptosis upon detachment.

Mechanistic studies in ovarian cancer revealed that miR-200c (Cittelly et al., 2012) restoration downregulated the core EMT transcription factors ZEB1 and ZEB2. This repression was sufficient to reverse the mesenchymal phenotype, restore E-cadherin expression, and re-sensitize cells to anoikis. In cervical cancer models, miR-525-5p (Chen and Liu, 2020) repressed metastasis by blocking the UBE2C/ZEB1/2 axis, thereby disrupting EMT-mediated survival and migration.

Research on papillary thyroid carcinoma (PTC) demonstrated distinct integrin-regulatory mechanisms. miR-363-3p (Pan et al., 2019) targeted Integrin α6 (ITGA6), severing the laminin-mediated signaling required for survival. Furthermore, miR-99a-3p (Gao et al., 2021) was shown to target the chaperone protein GRP94. The suppression of GRP94 prevented the proper folding and membrane localization of Integrin α2 , thereby disrupting cell-matrix interactions.

##### 3.2.1.1 Other malignancies

Epigenetic dysregulation is critical for melanoma cells to survive matrix detachment. Specifically, the upregulation of miR-29b/c directly repressed DNMT3A and DNMT3B. Similarly, overexpressed miR-203 and miR-26b further targeted and downregulated DNMT3B as well as MECP2, collectively driving DNA hypomethylation. In contrast, miR-26a dynamically regulated EZH2 in a stage-specific manner: it is initially downregulated in non-metastatic cells to facilitate early transformation via EZH2 accumulation, but is subsequently upregulated in metastatic cells to suppress EZH2 (Gasque Schoof et al., 2015). Furthermore, miR-214 (Penna et al., 2013) coordinated progression by upregulating ALCAM through the complex suppression of TFAP2 and miR-148b. The upregulation of ALCAM promoted cell aggregation, allowing cells to form tumor emboli and survive in suspension. For PCa, the miR-23b/-27b cluster (Rice et al., 2016; Ishteiwy et al., 2012) functioned as a metastasis suppressor by targeting Fascin-1 (FSCN1) and HIP1R, dismantling the invasive cytoskeletal machinery and membrane trafficking pathways. In Ewing’s sarcoma, miR-138 (Tanaka et al., 2016) directly repressed FAK, the central transducer of adhesion signals, sensitizing cells to detachment. Similarly, miR-1827 (Guo et al., 2020) in LUAD targeted Caveolin-1 (CAV1), which subsequently disrupted the FAK/Src signaling cascade. Finally, epigenetic silencing of miR-203 (Taube et al., 2013) across various carcinomas was identified as a prerequisite for EMT induction and stemness acquisition, Moreover, Bone Sialoprotein (BSP) (Thuong et al., 2024) in lung cancer was found to facilitate resistance by inhibiting miR-150-5p, thereby activating ERK/MMP-14 signaling to drive EMT. In viral-associated cancers, HPV-induced methylation (Wilting et al., 2016) silenced key miRNAs to promote anchorage independence, while KSHV-encoded miRNAs (Kieffer-Kwon et al., 2015) targeted Tropomyosin 1 (TPM1) to destabilize actin filaments in infected cells.

#### 3.2.2 Rewiring survival signaling and metabolic adaptation

Following the initial detachment, cells invariably encountered metabolic stress and signal deprivation. This phase represented a critical intermediate stage of compensation and adaptation. The literature revealed that miRNAs acted as central hubs that “hijacked” intracellular kinase cascades and reprogrammed metabolic networks. These adaptations ensured a continuous supply of energy (ATP) and the clearance of toxic byproducts (ROS), thereby maintaining cellular viability in a hostile, anchorage-free environment (Fu et al., 2022): Pro-Survival Kinase Cascades: miRNAs sustained the activation of the PI3K/AKT/mTOR axis, MAPK/ERK pathway, JAK/STAT3 signaling, and the Hippo/YAP axis to override death signals (D'Arcy, 2019); Metabolic Reprogramming: Mechanisms included the modulation of lipid metabolism and regulation of HIF−1α to adapt to hypoxia (Fulda, 2009); Stress Response Mechanisms: miRNAs promoted Protective Autophagy (targeting Beclin1, Atg5) aFignd managed oxidative stress through antioxidant regulators (e.g., Sestrins, SOD2) (Hanahan and Weinberg, 2011); TME Crosstalk: This involved intercellular communication via Exosomes and the modulation of growth factor receptors (IGF1R) or neurotrophic signaling (BDNF/TrkB).

Multiple signaling axes were identified in BC. miR-630 (Corcoran et al., 2014) targeted IGF1R, effectively blocking the upstream input to the PI3K/Akt pathway. miR-200b (Zhang X. et al., 2013) targeted the prolyl isomerase Pin1, crippling the Pin1-pERK axis and sensitizing cells to anoikis. Interestingly, miR-200a (Yu et al., 2013) targeted YAP1, highlighting a context-dependent role where YAP1 suppression paradoxically promoted metastasis. Metabolic adaptation was modulated by miR-6744-5p (Malagobadan et al., 2020), which targeted the enzyme NAT1, and miR-18a (Krutilina et al., 2014), which targeted HIF−1α to block hypoxic responses. Furthermore, miR-200c (Howe et al., 2012) disrupted an NF−κB -driven TrkB/NTF3 autocrine survival loop, while miR-181a (Polo-Generelo et al., 2022) was upregulated by TGF−β to promote BC metastasis via survival signaling.

In CRC, miRNA-503 (Noguchi et al., 2016) targeted the calcium-sensing receptor (CaSR), leading to the sustained activation of PI3K/AKT and ERK pathways. Of note, a context-dependent duality was observed regarding miR-145-5p (Cheng et al., 2022). While it functioned as a suppressor of EMT in early-stage disease, advanced CRC cells exploited high levels of miR-145-5p to enhance stem-like traits and anoikis resistance. This paradoxical effect was mediated by the modulation of the PI3K/AKT signaling cascade, where sustained pathway activation supported the resistant phenotype. For esophageal adenocarcinoma, miR-21 (Zhao et al., 2018) promoted resistance by simultaneously targeting PTEN (a negative regulator of PI3K) and PDCD4, while miR-26a (Zhang Y. F. et al., 2013) regulated the Rb1-E2F1 axis, linking cell cycle progression to survival.

Downregulation of miR-133a-3p (Tang et al., 2018) in PCa led to the hyperactivation of PI3K/AKT signaling. Additionally, epigenetic silencing of miR-132 (Formosa et al., 2013) by DNA methylation led to the upregulation of pro-survival genes. Furthermore, MTA1 (Cui et al., 2011) was shown to promote resistance by regulating FOXO3a, altering downstream apoptotic signaling, while miR-1247 (Taddei et al., 2019) was found to be downregulated by stromal signals, highlighting the critical role of TME-driven adaptation in this malignancy.

MiR-30a (Fu et al., 2018) promoted anoikis in HCC by targeting Beclin 1 and Atg5, thereby blocking protective autophagy, a key survival mechanism under stress. miR-25-5p (Liu et al., 2018) was transferred via tumor-derived exosomes to target RhoGDI in recipient HCC cells, activating Rho GTPases to drive motility. A sophisticated “disposal” mechanism was observed in BCa, where cells actively excreted the tumor-suppressive miR-23b (Ostenfeld et al., 2014) via RAB27B-dependent exosomes to maintain low intracellular levels and high resistance.

In ovarian cancer, miR-450a (Muys et al., 2019) targeted ODC1, disrupting polyamine homeostasis and energy metabolism, while miR-204 (112) targeted BDNF. In endometrial cancer, the interaction between the miR-200 family and Sestrin proteins (Kozak et al., 2019) was crucial; miR-200c overexpression decreased Sestrin1/3, dysregulating AMPK/mTORC1 signaling and sensitizing cells to metabolic stress.

In melanoma, miR-214 (Penna et al., 2011) contributed to progression by suppressing the transcription factor TFAP2C. Additionally, In endometrial cancer cell lines, the miR-200 family—mainly including miR-200a/b/c, miR-141, and miR-429—exhibits a significant inverse correlation with the expression of Sestrin proteins (SESN1/SESN2). As classical sensors of oxidative stress and key regulators of metabolic homeostasis, Sestrins attenuate energy and oxidative stress following loss of ECM attachment by inhibiting mTORC1 signaling, scavenging ROS, and activating AMPK, thereby suppressing the induction of anoikis (Zhu et al., 2020). In chondrosarcoma, Melatonin treatment (Nguyen et al., 2023) inhibited metastasis by enhancing miR-520f-3p production, which subsequently suppressed MMP7.

#### 3.2.3 Direct blockade of the apoptotic machinery

Regardless of upstream signaling, the decision to undergo anoikis was ultimately executed by the mitochondrial apoptotic pathway. Mechanisms in this category involved miRNAs that directly targeted the core executioners of cell death. By effectively “clamping” the apoptotic machinery, these miRNAs enforced a blockade on MOMP and enzymatic cleavage (Fu et al., 2022): The MOMP Rheostat (Bcl-2 Family): miRNAs shifted the balance towards survival by upregulating anti-apoptotic members (Bcl-2, Bcl-XL, Mcl-1) or directly silencing pro-apoptotic executioners (Bim, PUMA, Bax, Bak) (D’Arcy, 2019); Caspase Inhibition: Direct suppression of Caspase-3, Caspase-8, or Caspase-9 expression and activity was observed.

Exosomes derived from gemcitabine-resistant pancreatic cancer cells transferred malignant traits via the delivery of miR-222-3p (Wei et al., 2017). Upon internalization by recipient cells, this miRNA directly targeted and silenced the pro-apoptotic BH3-only protein PUMA. This suppression prevented MOMP and conferred a multi-drug resistant phenotype.

In BC, miR-181a (Taylor et al., 2013) directly targeted and silenced Bim, a key activator of Bax/Bak. Similarly, miR-223 (Pinatel et al., 2014) targeted the transcription factor FOXO1, thereby preventing the transcriptional induction of Bim. In melanoma models, miR-138-5p (da Cruz et al., 2021) suppressed p53, which subsequently reduced the transcription of downstream pro-apoptotic targets, including Bax and PUMA.

MiR-141 (Mak et al., 2017) in ovarian cancer indirectly upregulated the anti-apoptotic protein Survivin (BIRC5) via the KLF12/Sp1 axis. Survivin acts as a direct inhibitor of Caspase-9. In NSCLC, miR-451 (Wang et al., 2011) targeted the Macrophage Migration Inhibitory Factor (MIF), disrupting the autocrine loop that protected cells against Caspase activation.

A functional screen of small RNA libraries identified miR-30b and miR-30c (Moreno-Mateos et al., 2013) as key resistors of anoikis. These miRNAs were found to directly inhibit the intrinsic apoptotic pathway by reducing the expression of the executioner Caspase-3, preventing the final cleavage of cellular substrates.

#### 3.2.4 Anoikis-related signatures and prognostic landscapes

A significant body of research focused on the clinical and systemic implications of anoikis resistance. This section integrated studies that utilized high-throughput sequencing and bioinformatics to construct ARLs. This part highlighted the collective significance of anoikis-related molecules as biomarkers rather than isolated mechanistic effectors.

Chen et al. (2015) identified a miRNA cluster involving miR-7, miR-21, and miR-107 in HCC as contributors to HBx-induced progression and anoikis resistance. Tan et al. (2014) mapped miR-522 to a primate-specific regulatory network that collectively regulated adhesion and apoptosis genes to support survival. Yin et al. (2017) identified circUBAP2 as an oncogenic factor in lung cancer via functional screening, where its silencing significantly inhibited cell proliferation and invasion.

## 4 Pyroptosis

### 4.1 A brief overview of pyroptosis

As a form of pro-inflammatory RCD, pyroptosis was first formally proposed in 2001 (Cookson and Brennan, 2001). Mediated by caspase-dependent pore formation, pyroptosis involved cellular swelling, plasma membrane rupture, and efflux of cytoplasmic contents, inducing subsequent inflammation (Fink and Cookson, 2006; Rao et al., 2022). Gasdermin D (GSDMD) protein was the key executor of pyroptosis due to its pore-forming function. GSDMD could be cleaved by caspases, releasing amino-terminal gasdermin-N domain (GSDMD-N) and carboxy-terminal gasdermin-C domain (GSDMD-C). GSDMD-N then bound cell membranes and formed permeability pores on membrane, inducing pyroptosis (Shi et al., 2015; Sborgi et al., 2016). This process was recapitulated in GSDMA/B/C/E, which were mediated by different caspases and other factors (Chen and Broz, 2024). With the increasing breadth of research, various substances ranging from natural product to chemical compounds have been found to regulate pyroptosis in tumors (Xi et al., 2024; Wang G. R. et al., 2024; Shi et al., 2022). Studies based on pyroptosis-related immunotherapy and nano-drug delivery have also emerged (Zhu et al., 2025), and many pyroptosis-based prognostic models have been constructed to predict immune response and tumor progression (Xu et al., 2024; Zhou et al., 2022).

Several major signaling pathways of pyroptosis were as follows (Fu et al., 2022). Canonical pathway: pathogen-associated molecular patterns (PAMPs) and damage-associated molecular patterns (DAMPs) were recognized by pattern recognition receptors (PRRs) such as NLRP1, NLRP3, NLRC4, AIM2, PYRIN, etc. (Rao et al., 2022; Wu et al., 2010). The PRRs then bound to pro-caspase-1 and the adaptor protein apoptosis-associated speck-like protein (ASC), assembling inflammasomes, which subsequently activated caspase-1 to cleave GSDMD and induced pyroptosis. Meanwhile, caspase-1 cleaved inactive interleukin-1β (IL-1β) and interleukin-18 (IL-18) precursors, releasing the active ones to amplify inflammation (Man and Kanneganti, 2016; Miao et al., 2010) (D’Arcy, 2019). Non-canonical pathway: interacted with lipopolysaccharide (LPS) from Gram-negative bacteria, caspase-11 in mice or caspase-4/5 in humans were activated and then cleaved GSDMD (Kayagaki et al., 2015; Shi et al., 2014). The K^+^ efflux driven by GSDMD-N pores also promoted the activation of NLRP3 inflammasome and caspase-1, triggering a cascade which enhanced the release of IL-1β and IL-18 (Rühl and Broz, 2015; Fulda, 2009). The caspase-3/8-mediated pathway: with the stimulation by tumor necrosis factor (TNF), GSDME was cleaved by caspase-3 and caspase-8. GSDME-N then caused pores formation and triggered pyroptosis (Wu et al., 2023). Activated by different factors, caspase-8 also directly cleaved GSDMC and GSDMD (Zhang et al., 2021; Orning et al., 2018; Hanahan and Weinberg, 2011). The granzymes-mediated pathway: released by certain immune cells, granzyme A (GZMA) and granzyme B (GZMB) (Zhou et al., 2020; Zhang Z. et al., 2020) have been found to respectively cleave GSDMB and GSDME, forming pores in the membrane, leading to pyroptosis.

### 4.2 Interaction between pyroptosis and MiRNAs in cancers

**FIGURE 4:**
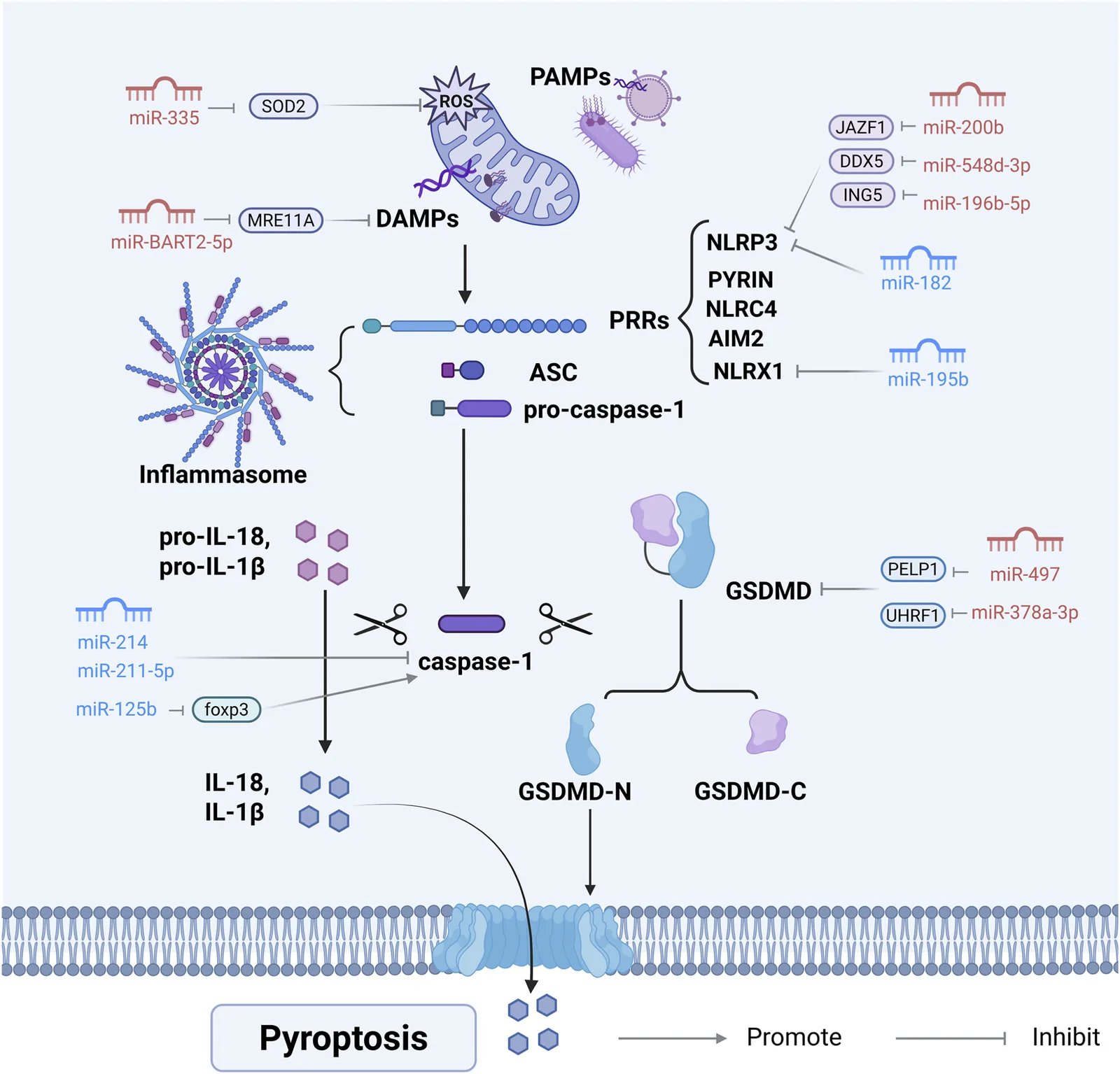
Regulation mediated by MiRNAs in pyroptosis. In the canonical pathway, DAMPs or ROS are recognized by PRRs, leading to the assembly of inflammasomes. The assembled inflammasome activates caspase-1, which cleaves GSDMD to form membrane pores. Within this cascade, miRNAs target upstream stimulation, inflammasome components, caspase-1 and GSDMD to regulate pyroptosis.

Diverse miRNAs have been shown to regulate pyroptosis, thereby affecting cancer development. These miRNAs exhibited aberrant expression in tumor cells compared to normal cells. By targeting various factors in different pathways, such as GSDMs, caspases, inflammasomes and the danger signals, miRNAs either inhibited or promoted pyroptosis, thereby influencing tumor progression and drug resistance in different cancers (Table 3; Figure 4).

**TABLE 3:** MiRNAs modulating pyroptosis in cancers.

MiRNA name	Cancer	Target/Regulatory axis	Effect on pyroptosis	References
miR-378a-3p	Esophageal Adenocarcinoma	UHRF1	Promote	Wang J. et al. (2025)
miR-497	Esophageal Squamous Cell Carcinoma	PELP1	Promote	Wang et al. (2019)
miR-214	Glioma	Caspase-1	Inhibit	Jiang et al. (2017)
miR-211-5p	Melanoma	GNA15/caspase-1	Inhibit	Zeng et al. (2023)
miRNA-125b	Nasopharyngeal Carcinoma	foxp3/caspase-1	Inhibit	Wang Y. et al. (2021)
miRNA-195	Neuroblastoma	NLRX1	Inhibit	Zhu et al. (2021)
miR-196b-5p	Non-Small Cell Lung Cancer	ING5	Promote	Zhang Z. et al. (2024)
miRNA-548d-3p	Non-Small Cell Lung Cancer	DDX5/JAK2 DDX5/STAT3	Promote	Liu et al. (2025)
miR-200b	Triple-Negative Breast Cancer; Luminal A Breast Cancer	JAZF1/NF-κB	Promote	Wang J. G. et al. (2021)
miR-182	Triple-Negative Breast Cancer; Luminal A Breast Cancer	NLRP3	Inhibit	Wan et al. (2023)
miR-335	Triple-Negative Breast Cancer; Luminal A Breast Cancer	SOD2	Promote	Wang et al. (2023)

#### 4.2.1 MiRNAs modulate pyroptosis by influencing inflammasomes

As cytosolic multimeric innate immune signaling complexes, inflammasomes have emerged as key mediators in the different pathways of pyroptosis. They were typically composed of a PRR like NLRP1/2/3 or AIM2, an adaptor protein like ASC, and an effector enzyme like pro-caspase. The inflammasomes were assembled in response to infection or cellular damage. Upon activation, they cleaved pro-caspases into their active form. The active caspases then cleaved pro-IL-1β, pro-IL-18 and GSDMs to initiate the pore-forming phase of pyroptosis (Wu et al., 2010; Ouyang et al., 2023). The formation and activation of the inflammasomes could serve as a target for miRNAs.

By targeting upstream regulators of NLRP3, some miRNAs activated the NLRP3 inflammasome, ultimately leading to the induction of pyroptosis. In the context of BC, miR-200b directly decreased JAZF1 expression. This then activated NF-κB signaling, which functioned as a transcriptional priming signal for the NLRP3 inflammasome, ultimately leading to pyroptotic cell death (Wang J. G. et al., 2021). Beyond this, miRNA was implicated in chemoresistance of BC. In paclitaxel (PTX) and DDP-resistant BC cell lines, high expression of signal transducer and activator of transcription 5 (STAT5) and miR-182 was correlated with reduced pyroptosis and decreased apoptotic rate. STAT5 directly bound to the promoter region of miR-182, promoting its transcription. Increased miR-182 subsequently decreased NLRP3 transcriptional level, reducing the formation of NLRP3 inflammasome, which suppressed the pyroptotic pathway (Wan et al., 2023).

In NSCLC, miRNA-548d-3p was capable of enhancing pyroptosis *in vivo* and *in vitro*. By binding to 3′-UTR of DDX5 mRNA, miR-548d-3p directly downregulated the RNA helicase DDX5, which was reported as a suppressor of NLRP3 inflammasome activation. DDX5 was further demonstrated to suppress activation of the NLRP3/Caspase-1/GSDMD pyroptotic pathway via inhibiting the phosphorylation of JAK2 and STAT3 (Liu et al., 2025). Another study revealed that miR-196b-5p was enriched in NSCLC-derived exosomes and transferred to T cells upon internalization. In T cells, miR-196b-5p suppressed the expression of ING5, resulting in pyroptosis (Zhang Z. et al., 2024). ING5 was found to inhibit Akt/NF-κB, which influenced the expression of NLRP3 (Zheng et al., 2022). In contrast to studies focusing on tumor cells, this study centered on processes occurring within T cells and revealed a novel strategy of tumor immune evasion. To restore anti-tumor immune responses, blocking tumor-derived exosomal miR-196b-5p to prevent T cell pyroptosis might be a potential strategy. In addition to NLRP3, miRNAs could also regulate other types of inflammasomes. A study verified that miR-195 bound to the 3′UTR of NLRX1 and negatively regulated its expression, leading to the inhibition of NLRX1 inflammasome assembly and subsequent suppression of Enterovirus A71 (EV-A71)-induced pyroptosis (Zhu et al., 2021). Although this study utilized human neuroblastoma (NB) cell line SH-SY5Y as a neuronal model to investigate EV-A71 neuropathogenesis, SH-SY5Y cells are intrinsically malignant and retain tumorigenic properties. Meanwhile, miR-195 has been well documented to participate in the regulation of various cancers (Yu W. et al., 2018). Therefore, the pyroptotic pathway mediated by the miR-195/NLRX1 axis revealed in this study may also function within the tumor microenvironment. MiR-195 may serve as a potential therapeutic target for tumors.

#### 4.2.2 MiRNAs modulate pyroptosis by influencing the caspases

As evolutionarily conserved cysteine proteases which cleaved substrates after aspartic acid residues, caspases family were key mediators of pyroptosis. Caspase-1, activated by inflammasomes, as well as caspase-4/5/11, activated by direct cytosolic LPS, cleaved GSDMD to trigger pore formation. Furthermore, apoptotic caspases could induce pyroptosis in certain contexts, with caspase-3 cleaving GSDME and caspase-8 cleaving GSDMC or GSDMD (Dho et al., 2025). Several miRNAs functioned as suppressors of caspases.

In melanoma, by targeting gene GNA15, exosomal miR-211-5p enhanced the metastasis competency of low-metastatic melanoma tumor cells through modulating immune responses, suppressing pyroptosis and promoting glycolysis. For pyroptosis, GNA15 facilitated the pro-metastatic function of exosomal miR-211-5p. It was validated that miR-211-5p downregulated caspase-1 and caspase-4, thereby inhibiting pyroptosis (Zeng et al., 2023). Additionally, tanshinone IIA considerably inhibited the proliferation of NPC cell line by promoting pyroptosis. As the target of tanshinone IIA, miRNA-125b was downregulated, which resulted in increased expression of its target gene foxp3. The upregulation of foxp3 executed pyroptosis in the pathway modulated by caspase-1, as evidenced by the decrease in caspase-1 and cleaved GSDMD when silencing foxp3 (Wang Y. et al., 2021). Moreover, compared with normal brain tissue, glioma tissues and cell lines exhibited lower miR-214 and higher caspase-1. Caspase-1 was identified to be downregulated by miR-214 through luciferase assays. Thus miR-214 regulated pyroptosis by targeting caspase-1 (Jiang et al., 2017).

#### 4.2.3 MiRNAs modulate pyroptosis by influencing the GSDMD

The gasdermins (GSDMs) were a family of pore-forming proteins that mediated pyroptosis by permeabilizing the plasma membrane. Apart from PJVK, the other five members (GSDMA, GSDMB, GSDMC, GSDMD and GSDME) shared a common structure consisting of three protein domains: a pore-forming N-terminal domain (GSDM-N), a linker, and a C-terminal domain (GSDM-C) (Chen and Broz, 2024). A few miRNAs could affect the expression or the cleavage of GSDMD according to recent studies.

Some drugs or inducers trigger pyroptosis by modulating miRNAs, thereby exerting their anti-cancer effects. In esophageal adenocarcinoma (EAC), upregulation of miR-378a-3p inhibited the proliferation of EAC cells, while suppression of ubiquitin-like with PHD and ring finger domains 1 (UHRF1) expression significantly inhibited their metastasis. The conversion of GSDMD proteins to GSDMD-N was facilitated by the low expression of UHRF1, which was manipulated by globular adiponectin (gAD) -induced upregulation of miR-378a-3p. This process was also validated *in vivo* (Wang J. et al., 2025). As for esophageal squamous cell cancer (ESCC), metformin has been demonstrated to induce pyroptosis. Mechanistically, metformin treatment upregulated miR-497, which in turn downregulated the scaffolding oncogene PELP1. Since PELP1 inhibited GSDMD cleavage and suppresses GSDMD translocation from cytoplasm to membrane, its downregulation ultimately promoted pyroptosis (Wang et al., 2019).

#### 4.2.4 MiRNAs modulate pyroptosis by influencing the upstream stimulation

Oxidative stress, characterized by the excessive accumulation of ROS, served as a critical trigger for pyroptosis through its capacity to activate the NLRP3 inflammasome (Wang J. et al., 2024). Notably, ROS accumulation was associated with the release of mitochondrial DAMPs (Koenig and Buskiewicz-Koenig, 2022). DAMPs functioned as signaling molecules which activated pyroptosis in various circumstances. For instance, released mtDNA or cytosolic DNA damage signals were sensed by PRRs, leading to inflammasome activation and subsequent pyroptosis (Broz, 2025). MiRNAs could modulate the activation of pyroptosis at the upstream level by affecting oxidative stress processes and cellular damage.

Through the regulation of mitochondrial enzymes or disruptive factors, miRNAs play distinct roles in triggering pyroptosis. Evidence from BC models indicated that the zinc finger protein 148 downregulated miR-335, consequently elevating the level of Superoxide dismutase 2 (SOD2). SOD2 then reduced ROS accumulation, which in turn suppressed oxidative stress-mediated pyroptosis (Wang et al., 2023). In NPC, tumor-secreted exosomes delivered miR-BART2-5p to vascular endothelial cells. This miRNA then downregulates MRE11A to trigger pyroptosis, increasing vascular permeability, and ultimately facilitating tumor cell extravasation and metastasis (Chen X. et al., 2025). MRE11A deficiency caused the release of mtDNA into the cytosol, which served as a DAMP to activate inflammasome assembly (Li et al., 2019). This intercellular communication warrants particular attention. Beyond miRNAs’ role in tumor cells, regulating pyroptosis in stromal and endothelial cells within the tumor microenvironment is critical for suppressing metastasis and improving therapeutic outcomes.

## 5 Concluding remarks and future perspectives

RCD functions as a barrier against uncontrolled proliferation, yet cancer cells frequently evade or reprogram it to acquire survival advantages and therapeutic resistance. As summarized in this review, miRNAs have emerged as pivotal modulators of ferroptosis, anoikis, and pyroptosis by modulating iron homeostasis, lipid peroxidation, adhesion-dependent signaling, and the inflammasome cascades. MiRNAs also exert substantial effects on other forms of RCD. For instance, miR-133a-5p facilitates cuproptosis through targeting of ATP7B (Ren et al., 2025). Besides, miR-1-3p suppresses mitophagy in ESCC by targeting SLC7A11 (Zhen et al., 2025). SLC7A11 also plays an important role ferroptosis and disulfidptosis (Li et al., 2023; Liu et al., 2023). What’s more, the miR-148/152 family facilitates cancer evasion by repressing RIPK1, thereby inhibiting necroptotic pathways (Li et al., 2026). Collectively, given the expanding classification of RCD pathways, systematic investigation and integrative reviews of miRNA-mediated regulation of RCD would be indispensable for deciphering the complexity of tumor survival mechanisms and developing multi-pronged therapeutic strategies.

Just as some compounds can simultaneously regulate several types of RCDs (Feng et al., 2024), certain miRNAs also exhibit such multifunctional regulatory properties. For instance, by modulating different downstream targets, miR-522 and miR-9 serve as concurrent inhibitors of both ferroptosis and anoikis (Zhang H. et al., 2020; Zhang et al., 2018; Liu et al., 2017a; Tan et al., 2014). MiR-214 inhibits pyroptosis and anoikis whereas miR-497 and miR-200b lead to opposite results (Yu et al., 2023; Penna et al., 2013; Zhang X. et al., 2013; Pinatel et al., 2014; Wang J. G. et al., 2021; Jiang et al., 2017; Wang et al., 2019). Due to the distinct metabolic demands and signaling networks of different tumor microenvironments, the same miRNA might regulate different types of RCD in a tumor-specific manner. The miRNA–RCD framework developed in this review introduces a previously absent perspective for RNA-based cancer therapeutics, offering a biologically grounded foundation for target prioritization, rational combination strategies, and patient stratification. The stability of miRNAs in body fluids supports their utility as biomarkers for treatment response and emerging resistance (He et al., 2020), whereas their druggability enables precise manipulation of RCD susceptibility through antisense oligonucleotides (ASOs), siRNAs, miRNA mimics, or CRISPR-based interventions (Tang et al., 2021; Kara et al., 2022).

Despite these promising findings, the clinical translation of miRNA-based regulation of RCD still faces substantial challenges that need to be addressed. The disproportionate control across pathways underscores these miRNAs’ potential as multidimensional therapeutic targets and biomarkers, but the clinical translation necessitates rigorous characterization of tumor-specific metabolic and signaling contexts (Zhang W. et al., 2020). For example, miR-200c represents a prime therapeutic target for TNBC and reversing paclitaxel resistance, whereas the context-dependent dual role of miR-200a under oxidative stress warrants caution in the use of its agonists (Howe et al., 2012; Yu S. J. et al., 2018). Therapeutic modulation of autophagy must similarly account for the miRNA landscape: in tumors with low miR-26a, autophagy is protumorigenic and should be suppressed, whereas in other contexts it may act as a cell death mechanism (Zhang et al., 2015; Zhang Y. F. et al., 2013). Furthermore, exosomal export of miR-23b by cancer cells (Ostenfeld et al., 2014) highlights that effective therapeutic design requires not only ensuring intracellular delivery but also preventing exosome-mediated export of the miRNA. For biomarkers, recent bioinformatic studies indicate that single-gene markers are often insufficient to predict complex metastatic phenotypes. Future research should focus on developing multi-gene prognostic models incorporating these key miRNAs, potentially extending to multiple RCD axes. For drug platforms and delivery technologies, approved RNA therapeutics continue to center on siRNA and ASO platforms rather than miRNA-based modalities, highlighting a substantial gap between mechanistic research and translational implementation. The challenges arise from the multi-target nature of miRNAs and common limitations in RNA delivery, tissue specificity, safety evaluation, and long-term toxicity (Winkle et al., 2021). As RNA drug platforms, nanodelivery systems, and personalized treatment approaches continue to advance (Li et al., 2021), strategies harnessing miRNA-mediated control of RCD are positioned to transition from mechanistic insight toward clinical application (Xue et al., 2022).

Taken together, these findings position miRNAs as a unifying regulatory layer that integrates multiple programmed cell death modalities and fundamentally shapes tumor adaptability, therapeutic sensitivity, and metastatic potential. By orchestrating ferroptosis, anoikis, and pyroptosis in a context-dependent manner, miRNAs enable cancer cells to fine-tune survival strategies under metabolic stress, immune pressure, and treatment exposure. Their stability, druggability, and pathway-spanning influence underscore their promise as multidimensional biomarkers and actionable therapeutic targets. Additionally, the complexity of their network architecture—together with tumor-specific metabolic and microenvironmental heterogeneity—highlights the need for systematic, multi-omics-driven mapping of miRNA–RCD interactions and rigorous translational frameworks. As RNA-based drug platforms and delivery technologies continue to advance, leveraging miRNA-mediated control of cell death holds substantial potential to overcome resistance, refine patient stratification, and ultimately guide the development of next-generation precision cancer therapies.
